# Reviews of Dental Literature

**Published:** 1905-05

**Authors:** 


### Reviews of Dental Literature.

Highest Orthodontia. 1. Facial Beauty; 2. Dental Antagonism. By John Nutting Farrar, M.D., D.D.S., New York City.[1]

Mr. President, Ladies, and Members of the Alumni of the New York College of Dentistry,—My subject this evening will be "facial beauty" and "dental antagonism." In other words, I shall speak upon that which I regard should be the highest desideratum in operations for the correction of irregular teeth.

The question is, should mastication of food, without much regard to facial beauty, be the main aim, or should it be, efficient mastication of food with special regard to improvement of facial beauty? For many years my systematic, earnest efforts have by tongue and pen been the pointing out of the steps that have been taken from the early ideas to the present time, the gradual but not regular progress of the art and science of dentistry, especially in the branch for correction of irregularities of natural teeth; showing at the same time a glimpse into its future, for I believe there is yet a great deal to be done before the best results will be accomplished from education along this line.

The question of extraction would naturally come in here for discussion, but as I have already somewhat exhaustively treated this branch of the subject in Chapters LXV., LXVI., LXVII., pp. 681 to 711, in my Volume I., upon "The Irregularities of the Teeth and their Correction," I shall only again incidentally refer to the subject in this lecture. The subject of my lecture, "Facial Beauty *versus* Dental Antagonism," is no new phase of my teaching,

---

[1] Lecture delivered before the New York Dental College Alumni, January 18, 1905.

either orally or in print; my present object shall be to reach out into broader and loftier branches upon the subject of correction of irregular teeth, pointing out some of the errors of the retrogressive teachings, which I believe if followed will be the entering wedge to the destruction of our highest and noblest branch of dentistry.

Theory of perfection in the correction of irregularities of the teeth, and theory of perfection in treatment in general medicine, are written upon quite similar lines; both are to serve simply as general guides in practice. *There is no one best rule* for all cases. The vicissitudes of circumstances, conditions, and environments must be considered, or results may be unsatisfactory. This variation may be partly illustrated by the difference between hospital practice and family practice, whether it be in medicine or dentistry. Many things that can be forced in hospital practice cannot be forced in family practice, "*subject*" in one, "*independent personality*" in the other; a treatment in the former case that may be satisfactory to the patient, may not be satisfactory to the patient in the latter. Again, the matter of idiosyncrasy, diathesis, mental characteristics, as well as financial and social circumstances, should be taken into consideration. Some heads of families have proper ideas of their own; such persons are not always easily induced to change their views; especially is this true if opposed by illogical presentations.

It is often said that clear knowledge of anatomy is important to the physician and the general surgeon in order to enable him to be thoroughly efficient; but this is no more true of physicians and surgeons than that clear knowledge of anatomy and dental etiology (in which proper antagonism is a factor) is important to the dentist. While the general surgeon needs clear knowledge of the relative position of the muscles, nerves, and blood-vessels, and their relation to the osseous frame, so does a regulator of teeth need clear knowledge of the relations of the different parts of each jaw and the relation of the jaw-bones to the other bones of the head; nor is this equipment of education sufficient without a clear knowledge of anatomy of the soft tissues, and knowledge, in addition, of physiology, and the changes that may take place from physiological conditions to pathological conditions of the tissues during operations.

The regulator of teeth thus prepared will meet more easily the difficulties that may result from operations. But as important as all this knowledge, together with a knowledge of the development of the teeth, and their proper antagonism may be, it would not be sufficient to constitute a first-class education (for a regulator), without an equally clear knowledge of the laws of mechanics, in order to take advantage of the above-mentioned knowledge. Furthermore the dentist should understand the rules of proportion, of the different parts of the human face and head, as formulated by recognized great sculptors and painters, and also a knowledge of the differences between the several classes of human faces.

As there are several types of human faces and heads, and as there are as many types of bones of the heads, each head having its own form of jaw-bones, an effort to establish, in this enlightened age (regarding knowledge of the evolutionary laws), a geometrically formed typical human jaw, from one selected from many, that will constitute an *Adamic type for the whole human race,* may seem strange; but it does not seem strange that artists should attempt to classify beautiful human forms. But while all the classes of the most beautiful forms, as a whole, may be counted by less than the digits of two hands, the different classes of the human head would be greater in number, and the number of forms of heads that might be classified would not be equal to the classes of the various shades of beautiful faces belonging to those heads. For illustration, the heads may be classed, 1, the round or bullet form; 2, the square head; 3, the vertically long but broad head; 4, the long but thin head; 5, the oval head; 6, the egg form, with the smaller end down; and 7, the egg form, with the larger end down (varieties of the oval). Then there is the face with a Greek nose, the Roman nose, the hawkbill nose, the pug nose, the African nose, the ape nose, etc. Then there are the noses of the more beautiful forms of faces; noses the size and form of which harmonize with the other facial features, eyes, mouth, chin, and lips. Then there are the faces beautiful in form, but cold and forbidding; and those that are not so beautiful in form, but otherwise charmingly beautiful by intellectual scintillations shown in the expression through an animated face.

There are so many phases of my subject I cannot, in the limited time of this evening, go over all, and if I repeat, and apparently recross some tracks of my argument, it will be only to fasten the essential points in your memories. In dentistry much is said of dental antagonism, because much of success depends upon proper knowledge of the subject. In nature we find that dental antagonism is similar, but not at all uniform. But perfection, though rare, is sometimes found, and, rare as it is, the *perfect* should serve as a general guide, but not an absolute guide.

In the winter of 1864–65, Professor J. H. McQuillen gave a course of "special lectures" upon the subject which he denominated "Harmony of Antagonism." It was my good fortune to hear the entire course of these remarkable lectures upon the importance of proper antagonism. Not only was the "interlocking" or "break-joint" association of teeth shown, but the line of actual contact in normal occlusion of the two arches was clearly illustrated. Other persons have since that time treated in print the same subject, but none have presented it more clearly and interestingly than did McQuillen.

While abnormal antagonism of the teeth is often the result of inherited differences between the size of the jaw and the size of the teeth of the parents (immediate and remote), extraction of deciduous teeth at the wrong time and the wearing away of adult teeth by use, causing them to unduly press and slide upon each other, are also causes of irregularities. Then, again, that class which is denominated protruding teeth (generally the upper anterior teeth) may or may not be the result of dental antagonism.

Protruding upper teeth may be caused by inheritance or by thumb- and finger-sucking; but my experience teaches me that by far the majority of cases are caused by too long lower anterior teeth and too short side teeth; the latter being largely caused by the sag in the sides of the lower alveolar ridge; altogether causing too much pressure upon the posterior walls of the anterior upper teeth. But some of the major cases may be apparent only, caused by too short lower jaw, and others are indirectly the result of "mouth-breathing," caused by nasal obstruction, that tempt the jaws to remain apart most of the time in order to breathe easily, leaving the buccal muscles to act with undue power upon the side teeth, narrowing the arch and pushing the anterior teeth forward. Where mouth-breathing is continuous, and the size of the teeth and the size of the dental arches do not harmonize, and the teeth are also jumbled, the arches, that thereby become too narrow, may require widening. But if the patient's head is narrow (from side to side), the degree of widening should be carefully considered; especially is this so if the greatest improvement of the face is to be accomplished.

While there is *no one* best rule for regulating all cases, there is generally *one best way* of accomplishing the highest result *in a given case.* The highest aim in this branch of dentistry should be to accomplish not only the greatest possible degree of beauty in facial form, but also an improvement in mental expression, through the form. Movers of teeth there may be many, but moulders of the face, to the highest possible benefit, there are not so many. Those of the latter class may be regarded as the true artists in this kind of mobile surgery.

While efficient antagonism contributes largely to proper mastication, and such mastication contributes to proper digestion, that may lead to healthy complexion; *proper food* and *regular habits* have quite as much to do with it. While the regulator should give great attention to antagonism, he may fail in public and in professional estimation to have the reputation of mastership if he does not also give the greater attention to improvement in facial beauty.

To understand how to bring about *efficient* antagonism of the teeth it is not only important to enable proper correction, but it is also necessary to prevent recurrences of irregularities when once corrected.—*Items of Interest.*

(To be continued.)

ACTINOMYCOSIS OF THE MOUTH AND FACE, WITH THE REPORT OF TEN CASES. By Thomas H. Kellock, M.A., F.R.C.S., etc.

MR. PRESIDENT AND GENTLEMEN,—It was with a good deal of pleasure that I acceded to a request that I should bring before your Society some of the cases of actinomycosis which it has been my fortune to meet with recently in the out-patient department at the Middlesex Hospital, and I am glad to have been able to secure the attendance here this evening of a few patients suffering from this affection in the regions about the mouth, who serve, I hope, to illustrate fairly well the affection in different stages, and also the points connected with the clinical aspect of the disease, to which I would like to call attention. Whether it is that this affection is rapidly becoming more common, or that interest in the subject has caused me to make more careful investigations in probable cases, and thereby recognize it more frequently, I am not sure; one or the other of these must have happened, for otherwise it would seem strange that relatively so many cases of what has generally been looked upon as a rare affection should have occurred in one out-patient department, and that not an exceptionally large one. As I shall presently hope to show, the confirmation of the diagnosis is not by any means always an easy matter, and this fact may account for many cases having been overlooked. On the other hand, it is difficult to understand what became of such cases as those I am showing, if they were not subjected to the treatment which, as far as we are aware, is the only one that has the effect of arresting or curing the disease, which is generally a progressive one, and one that shows little tendency toward spontaneous cure; it is curious, too, as going to show that the affection is more common than formerly, that there should have appeared in quite a recent number of the *Lancet* (October 29, 1904), the reports of three cases of this kind, calling forth from the editor an able leading article on the subject.

It is easily within my memory when actinomycosis was included with such rare affections as hydatid disease and filaria sanguinis, and hardly given a place when discussing the differential. diagnosis of tumors and abscesses about the face, jaws, or other parts of the body. The occurrence, however, of so many cases among the out-patients has compelled me to include it in my teaching as a disease that is by no means rare at the present time, and I think its importance, both from the point of view of the patient and of the surgeon, justifies this.

I may, perhaps, be allowed to call attention to a few points in connection with the pathology of this affection. The name actinomycosis was given it with the idea that the fungus, which is the specific cause, could be recognized, microscopically, by its "starred" or "ray-like" appearance. We now know that there are several varieties of fungus or streptothrix that can act as the cause,—fungi which are found in twisted masses of mycelium, and only really visible when suitably stained, the peculiar ray formation being due to refraction of light around particularly disposed masses of these. This it is important to remember, for in attempting to make a diagnosis it is often only an unstained specimen of pus or granulation tissue that is for the moment available, and it by no means follows that this ray formation will be seen in all cases, and one has often failed to find it in cases in which subsequent staining has demonstrated the presence of a streptothrix. Another important point to bear in mind in this connection is that this ray formation may be broken up, and the evidence of it visible in the unstained specimen looking like minute elongated fragments of glass scattered about among the pus cells, and I have once or twice been able to recognize these in specimens where the ray formation was not seen. The difficulty of recognizing the affection from an unstained specimen of the discharge is greatly increased in cases where acute suppuration has supervened; in these cases difficulty is often also experienced even after staining, and I have known one or two cases of this sort where it was only after repeated examination of the pus that streptothrix was found to be stained. This same difficulty is, we know, experienced in demonstrating in similar cases the bacillus of tuberculosis, an organism to which it would seem these streptothrices have in many respects a very close resemblance.

The filaments of these streptothrices stain well by Gram's method, becoming of a red-blue color; they vary somewhat in size and arrangement, but are alike in their twistings and convolutions and in the way in which they stain by this method. The fungus was formerly supposed to flourish almost exclusively on straw or hay, and, as you will remember, the chewing of them by man or beast was looked upon as the usual mode of its entry into the body; the occurrrence, however, of this affection among people of many and varied occupations and modes of life, even as instanced by the cases I am showing to-night, seems to indicate that the organism has, at the present time at any rate, a much wider distribution than was formerly supposed.

To turn now to the clinical aspect of the affection, which must be that of chief interest to us as dental or general surgeons, it is fair, I think to state that experience shows that, in by far the larger number of cases, the organism enters the body by means of the respiratory or alimentary tracts; isolated cases of its infection directly into the skin or subcutaneous tissues have been reported, just as in the case of tuberculosis, but these may be regarded as more or less rarities, and it is in the respiratory and alimentary tracts and in the organs which have a direct communication with them, that we find, at any rate, the primary infection.

With the affection as it is found in the lower parts of the alimentary and respiratory tracts such as the liver, vermiform appendix, pelvis, or lungs, we are not at present concerned, but

I should like to draw attention to one peculiarity of this affection as seen in the lungs, for it illustrates well what is seen in other parts of the body,—namely, its disposition to ignore anatomical boundaries, and spread beyond the limits of the organ primarily infected. It affects the lungs primarily in much the same way as does tuberculosis, but, unlike the latter, it has a great tendency not only to induce adhesions between the lung and the chest wall, but to invade the latter, and an actinomycotic affection of the lungs is often only recognized as such when it has produced an abscess pointing on the surface of the chest. This property is also seen in other organs, such as the salivary glands. Tuberculous affections, as a rule, are easily restrained, as it were, by such anatomical structures as fasciæ, synovial and serous membranes, and in this respect actinomycosis differs from them and more nearly approaches what is seen in malignant growths and tertiary syphilitic manifestations.

The organism having entered the mouth, carried in probably on some article of food or substance that is held in the mouth and chewed, there seem to be several ways in which it may find a resting-place where it may increase and multiply. It is doubtful if it ever affects a healthy, unbroken surface. Case 1, reported below, where the disease occurred in the substance of the tongue, may be an instance of this, but it is there quite easy to suppose a slight previous lesion, possibly only microscopic. Then there are those cases which we were formerly taught to consider the most common, namely, those in which the jaw is the part affected, the organism entering at a breach in the surface round a tooth, carious or otherwise, it matters not, so long as there is an open wound in the gum surrounding it. In its growth in this situation—always it seems in the *lower* jaw—it cultivates under the periosteum, and produces an abscess which generally, if not always, finds its way to the surface on the outside.

When this abscess bursts, or is opened, a track is formed leading down to a patch of bare bone of varying size. I say bare bone, in distinction to dead bone, because I think if these cases are actively treated it does not at all follow that any of the bone exposed by the lifting up of its periosteum by this affection need necessarily perish. If, however, the condition persists for some time, and especially if the surface of bone is exposed to a septic infection in addition to the other, necrosis will almost certainly follow—so like what is seen in tuberculous disease of bones in general.

The clinical history of these cases is important, and when a patient presents himself with a sinus leading down to a patch of bare bone in the lower jaw, with a history that it has persisted for some time, in spite of removal of the tooth supposed to be the cause of the trouble, and that throughout the whole course of his ailment—and to this point the greatest importance should be attached—there has been little or no pain, I generally begin to suspect that a streptothrix of some sort may be the cause, although, as I have stated before, the confirmation of the diagnosis may be a matter of considerable difficulty and only obtained after repeated examinations of what little discharge there may be coming from the sinus.

One more channel of entrance of the organism into the system remains to be mentioned. It is one which you will notice in the cases to be a common one, and yet it is one that hardly ever seems to have been remarked upon,—the salivary and mucous glands, which open into the mouth, and their ducts. You will notice among the cases instances of the affection starting apparently in the ducts of the parotid and submaxillary salivary glands, in the parotid gland itself, and, in one instance, in the sublingual gland. How it is that this particular organism should have the property of insinuating itself along these ducts in the reverse direction to the stream of the natural secretion, it is difficult to explain. May it be that at some particular time when it is present in the mouth, that there is some abnormal diminution of the secretion, and so the organism, having effected an entrance into the duct, is allowed to rest in peace or even find its way into the gland itself?

The fungus of actinomycosis does not seem to cause open ulcers, or to be taken up by the lymphatics and so affect the lymphatic glands in the neck, in the manner so common in tuberculosis, while a tuberculous affection of the lower jaw in adults, or of the salivary or mucous glands, appears to be extremely rare.

Something should be said as to the clinical characters of the abscesses in connection with this affection when they reach the surface; as a rule, they are chronic or subacute, for the time they take in forming seems to vary from a few days to some weeks. They first manifest themselves as a dusky hardness of the skin, unaccompanied, as a rule, by pain or rise of temperature, and only causing inconvenience when by infiltration they impair the function of muscles or other structures. One or more soft spots appear in the indurated area, and between these projecting soft spots the skin at the normal level appears to be drawn in, so giving rise to rather a typical appearance. If left alone these soft spots open spontaneously, and if several do so the swelling assumes a sort of honeycomb appearance. If they are incised, one is often almost disappointed at the very small quantity of pus that escapes compared with what one had expected from the size of the swelling. These soft spots do not, as a rule, communicate directly with each other, and thus several incisions may be required.

In the pus that escapes there can often be recognized the small, particularly spherical masses characteristic of the affection, sometimes bright yellow,—sulphur grains, as they have been called,—sometimes a dull yellowish-white, and sometimes again—an important fact to remember—very small and quite colorless. In the latter case they may be recognized by allowing some of the pus to flow slowly down the side of a test-tube, and in a good light the minute elevations caused by the colorless masses adhering to the glass can be seen.

If, as sometimes happens, when there is an open communication between the seat of the disease and the exterior of the body or the cavity of the mouth, acute suppuration is added, the case takes on a very different aspect, and the diagnosis becomes much more difficult; the patient is often very ill, suffering from all the effects of an ordinary septic abscess, and it is more than likely that the true nature of the affection will be overlooked until, the usual means having been adopted for the treatment of the acute abscess, the persistence of sinuses and the characteristic discharge from them give a clue as to the original cause of the trouble.

As to the treatment of this affection little has to be said, for it is simple; we cannot but look upon it as a communicable disease, and, therefore, prevention of its spread to other persons ought always to be in our minds; when once it is manifest, the proper course of treatment seems to be locally to incise the softened spots freely and scrape away all that is possible of the affected tissues, and generally to treat the patient with large doses of iodide of potassium, commencing with such a dose as fifteen grains three times a day, and increasing this rapidly till he is taking from fifty to sixty grains three or even four times in the twenty-four hours. It has been my custom to add a moderate quantity of arsenic, such as three or four minims of liquor arsenicalis; in the first instance, this was done to try and prevent the skin lesions which the large quantity of iodide might cause, but I cannot help thinking it has some effect on the disease itself. The prognosis under such treatment, at any rate when the affection occurs about the mouth or face, is certainly good, although it may need to be carried out continuously for a considerable time, and it is rather remarkable how comparatively little disfigurement or scarring results if the disease be eventually eradicated.

It only remains for me now to describe the specimens and cases I have to show. The former comprise—

1. Unstained specimens of pus, showing microscopically the characteristic "ray" formation.
2. Specimens showing microscopically a streptothrix stained by Gram's method.
3. Test-tube with pus containing characteristic yellowish-white granules.
4. Culture tube.

Before reading the notes of the patients who are in attendance this evening, I should like to narrate, briefly, those of two or three who have been under my care, but whom I could not bring this evening for various reasons. [The report of cases is omitted.]—*Transactions of the Odontological Society of Great Britain.*

A Brief Review of the Prevailing Theories of Immunity.—It is generally believed that the prevailing theories of immunity are extremely difficult to understand. This is due mainly to the fact that new technical terms have had to be introduced in order to avoid explanatory sentences each time a particular constituent is referred to. As each investigator has interpreted the functions of these various constituents in his own special way, he has deemed it necessary to rename the whole series, so that each substance actually found is now the bearer of many distinguishing appellations, to say nothing of the substances hypothetically introduced in the processes, and also given various names. Bereft of these obscuring features, the three prevailing theories, however, lose their complexity in a great measure.

*Metchnikoff's Phagocyte Theory.*—Metchnikoff, who began his investigations in 1865, terms "phagocyte" any cell capable of incorporating bacteria and of destroying them by a process of digestion. Haeckel (1862) saw leucocytes ingest indigo; Recklinghausen (1863) noted that pus-cells ingulfed cinnabar grains; Cohnheim (1867) saw leucocytes plunge their pseudopodia through vascular stomata and thus leave the vessels to become pus-cells. Even Metchnikoff's antagonists have recognized that the leprosy bacillus, the gonococcus, and other micro-organisms are always found in cells, while he has himself shown that they can ingest living anthrax bacilli. These organisms, having conquered the cells, passed out into the hanging drop, multiplied therein, and proved virulent on inoculation. As observed by Cantacuzène, anthrax bacilli injected in a vein of a rabbit's ear are destroyed in seven minutes in the liver, in eight minutes in the lungs, and in one hour in the spleen. The organisms that escape from one cell are seized by others, but if their multiplication is excessive they overpower the phagocytic leucocytes and invade the blood-stream.

Various observers, Pfeiffer, Büchner, and others, having demonstrated that the blood-serum and other body-fluids were likewise bactericidal, Metchnikoff ascribed this fact to the disintegration of phagocytes, the properties of these cells being thus imparted to the serum. As shown by Bordet and other French investigators, this property is due to two constituents of the plasma. The one (the specific immune body) circulates in the plasma, according to Metchnikoff, and resists a temperature as high as 100° C. The other, or "cytase," thought to be derived from disintegrated phagocytes, is destroyed by heating to 55° or 56° C. thirty minutes, and is considered by him as belonging to the category of trypsins.

Bulloch, in his review of the bacteriological side of the question of immunity for the section on pathology at the recent meeting of the British Medical Association, states that "thousands of facts point to the conclusion that our leucocytic-forming tissues are our great defensive organs against parasitic invasions," but that "the mystery is how the microbes are destroyed."

*Büchner's Alexins, or the Humoral Theory.*—The view that the serum is endowed with bactericidal powers has been ably defended by Pfeiffer, von Fodor, Nuttall, and others. Their labors led to a doctrine of which the late Professor Hans Büchner was the main exponent. Both he and Hankin isolated substances which they termed "alexins," and to these bodies they ascribed the power to confer immunity. They were also thought by Büchner to be derived from leucocytes (oxyphil) and kindred cells. Laschtscenko having isolated them from living leucocytes, the bactericidal action was thought by Büchner to be due to a proteolytic enzyme, similar to the ordinary digestive ferments. The "alexins" were found to lose their bactericidal power, however, when exposed thirty minutes to a temperature of 55° C.

Büchner recognized Metchnikoff's doctrine only to a certain extent, the phagocytes being considered by him as mere scavengers capable of removing from the blood various substances foreign to it, including bacteria weakened by the alexins. The cells were deemed incapable, however, of carrying on an active warfare against pathogenic micro-organisms in general. Grüber was also led by a large number of experiments to conclude that the actual destruction of bacteria in all animals, "whether actively or passively immunized," was due to Büchner's alexins, and that these alexins were "general protective substances entirely without specific action." According to Grüber, the bacterial cell walls were first made adhesive by the blood's agglutinins and thus became vulnerable to the destructive action of the alexins.

Büchner's alexins were soon found deficient in many particulars, but the many valuable paths his labors and those of pupils have opened have been utilized in the elaboration of other hypotheses.

*Ehrlich's Side Chain Theory.*—This theory, advanced by Ehrlich in 1897, aimed to harmonize the results so far reached in the study of natural and artificial immunity and particularly the formation and mode of action of the immunizing constituents of the blood-serum. Reduced to its simplest expression, Ehrlich's theory is, on the whole, not a complex one, and is based on recognized biological and chemical principles.

The body being composed of colonies of cells which differ from one another in their functional attributes, nerve-cells, muscle-cells, gland-cells, etc., we speak of the specific action of drugs, because each kind of cell has a special affinity for certain drugs. Thus, the cerebrospinal motor cells have affinity for strychnine, the cardiac muscle-cells for digitalis, the sweat and salivary gland-cells for jaborandi, etc. Now, toxins differ in no way from remedies in this particular; they also are specific owing to their cellular affinities, toxins being also taken up by specific cells. This specific action of toxins represents the foundation upon which Ehrlich's theory is poised.

The theory itself is based upon the mechanism of cell-nutrition in its relations to the mode of production of specific antitoxins, or bactericidal and antitoxic sera.

The complexity of cellular protoplasm is well known to biologists. Ehrlich assumes that each cell contains an active central nucleus (*leistungs Kern*) and of groups of molecules, or "side chains." These outer, or extranuclear, groups constitute a cardinal feature of the entire scheme, since each of them has for its function to receive (hence the term *receptor* given it by Ehrlich) the molecules of food-stuffs which perpetuate the cell's existence. These molecules Ehrlich terms *haptophores* (ἅπτειν, to touch; φερειν, to bring) owing to their identity as extrinsic carriers. A cell's nutrition is thus carried on through the affinity its "receptors," or "side chains," have for its haptophores.

Unfortunately, the receptors do not combine with these nutrient substances only; their affinity extends to substances which are, in a measure, their chemical analogues and which may include harmful bodies. Thus, a poison, a toxin, or a venom may contain the same elements and the same number of them as a nutrient molecule, or haptophore, the only difference between the latter and the pathogenic molecule being a different arrangement of their respective atoms (isomerism). The formula of turpentine, for instance, is $C_{10}H_{16}$, while that of oil of lemons is also $C_{10}H_{16}$; and yet their properties are quite dissimilar. The same may be said of ethyl formate, methyl acetate, and propionic acid, all of which have a molecular weight of 74, and the same formula,—*i.e.*, $C_3H_6O_2$. This makes it possible for a cell to take up (combined with its nutrient haptophores any number of isomeric non-nutritious bodies, and when one of these happens to be a toxic—thus forming what Ehrlich terms a *toxo*phore—the latter combines with the cell and, if sufficiently active qualitatively and quantitatively, may act destructively upon it.

If the toxic is not sufficiently active to destroy the cell or inhibit its functions, the latter seeks not only to rid itself of the poison, but also to protect itself against further aggressions from this poison and its isomers. Intoxication being due to a toxin, for instance, the toxin group of the haptophoric molecule attaches itself to the receptors and then reacts actively upon the cell proper. The receptors themselves being destroyed, the cell (in keeping with Weigert's theory of overproduction) not only replaces them by reproduction, but the process is so active that many more receptors are created than are required by the cell itself, and they accumulate in the blood and lymph. A very prominent feature of Ehrlich's theory appears in this connection,—viz., these surplus receptors preserve their affinity for their specific haptophores and therefore for any isomeric toxin that may combine with it. The receptors thus become the cell's protectors, since they capture, as it were, and hold the toxin before it can at all reach the cell proper, thus immunizing the latter against the poison. Diphtheria toxin, for instance, by exciting the cells for which it has specific affinity, causes these cells to liberate receptors in large quantities; these, by at once combining with the rest of the toxin in the blood-stream, curtail its morbid effects. *Antitoxin*, according to Ehrlich, owes its curative properties to the receptors (side chains) it contains.

The element of specificity, so evident in all immunizing processes, is easily explained by Ehrlich's conception. As the different cell-colonies constitute clearly defined and autonomous groups, each of which reacts under the influence of poisons which affect none of the other groups, a poison can only cause the production of one kind of receptors, and these receptors injected into another animal can only neutralize that one poison and its isomers. Again, the body being only vulnerable to the toxins with which its cells' haptophores can combine, it follows that Ehrlich's theory covers all diseases caused by toxins and the processes through which the body acquires immunity against them.

The destruction of the more solid pathogenic agents such as bacteria, yeast-cells, worn-out blood-cells, tissue débris, and other cellular structures require the aid of additional factors,—*i.e.*, cell "poisoning" or "killing" agents, which Ehrlich has termed *cytotoxins* or *lysins*. These bodies, according to Ehrlich, not only appear in the blood when useless (and therefore harmful) cellular materials are to be dissolved, but each body is essentially specific as a solvent. Thus, when blood-cells are to be destroyed, there appear in the blood-plasma *hæmo*lysins; when muscle-cells, *myo*lysins; when nerve-cells, *neuro*lysins; when kidney-cells, *nephro*toxins, etc. Each of these is active only in the case of one particular kind of cell and in that of no other. Bacteria, introduced into the circulation, also provoke the elaboration of specific antibodies, *bacterio*-lysins, and blood-plasma containing one of these is active only upon the bacillus which, injected into animals, caused its appearance in the blood. Serum obtained by injecting the typhoid bacillus, for instance, will destroy typhoid germs, but it will not dissolve the diphtheria bacillus or any other germ.

Two bodies or substances have been shown experimentally to be necessary in the process, in addition to the cellular material acted upon. One of these bodies is not affected by exposure to relatively high temperatures and has been termed *immune body* by Ehrlich, while the other, the *complement,* is destroyed when kept at a temperature of 55° C. thirty minutes. The immune body's rôle is to attach itself to the cells or bacteria to be dissolved and to act as a link (hence the name *amboceptor* also given it by Erhlich) between them and the complement, the latter having no affinity for the cellular elements. The complement being endowed, according to Ehrlich, with digestive powers, it is thus brought into contact with the cells and these are dissolved.

But how is the marked specificity witnessed in the destruction of these bacteria and other cellular *detritus* accounted for in this process? Ehrlich ascribes it exclusively to the immune body. Thus, if cholera bacilli be introduced into an animal the immune body that will appear in the serum obtained from that animal will attach itself to cholera bacilli only. This is because the combining group of the immune body thus obtained will fit only that particular species of micro-organism as a key does its lock. And this applies to all kinds of bacteria and cellular elements to be disposed of in the body fluids: they represent as many different locks, each of which requires its own particular key. Briefly, while the complement is the solvent, it cannot act upon bacteria or other cellular elements without being linked to them by one of the *great many* immune bodies which, according to Ehrlich and Morgenroth, may occur in the blood-serum.—*Editorial in Monthly Cyclopædia of Practical Medicine.*

COMPRESSED AIR IN DENTISTRY. By George Zederbaum, D.D.S., Charlotte, Michigan.[1]

IT is my purpose to bring before you the many advantageous uses of compressed air in the practice of dentistry.

We shall first consider the apparatus necessary to obtain and maintain a certain pressure of compressed air. In my opinion, the

[1] Read before the Michigan Dental Association, June 28, 1904.

best apparatus is the one described and illustrated by Dr. H. C. Register, of Philadelphia, in the April number of the *Dental Brief*, and on exhibition here with Sibley's goods. It consists of a small pump which operates by being connected by a pulley to an electric motor. When the motor is set in motion, the pump compresses the atmospheric air into a storage tank which, by means of an air-gauge, accurately registers the pressure of air stored. From the storage tank leads the outlet pipe, guarded by a suitable valve. Unfortunately the use of this outfit is limited to those who are located where a street electric current is obtainable. The other apparatus, and the one we all can have, is the one depending upon the water-supply, and this is available in almost all towns. No very high pressure is necessary. Here we have an "air pump which is attached to the city water-supply pipe, and which keeps a desired pressure of air in the storage tank automatically." A very small pump, such as I just described, will maintain from thirty to forty pounds pressure of air for a very long period. The manufacturers of these pumps claim that only one pound of pressure is lost by friction, that is, your compressed-air tank, when kept full, will register just one pound less than your water supply registers. The temperature of air so obtained is about 25° F. higher than that of the water at the same time. Either apparatus described may be had for some twenty-five or thirty dollars. They occupy but very little room, and in case of water-motor and tank, these can be stored in the cellar and air piped up to the most convenient point.

There are many other apparatuses on the market, some with hand- and others with foot-pumps, but I would have the same objections to these that I would have to a common rubber bulb atomizer,—namely, insufficient capacity and insufficient pressure of air for prolonged use. So much for the apparatus.

That compressed air is becoming more and more a necessity for us, and not merely a luxury, as many may suggest, one has to look into some of our valuable dental publications. There are quite a few of the thinking practitioners considering the installation of compressed-air outfits in their offices. It is only the other day that I read an advertisement in one of the dental journals of a modern dental office for sale; everything complete, electric engine, lathe, compressed-air outfit, etc. This is my sentiment exactly. No office is complete and up to date in every sense of the word without its compressed-air supply. Now let us consider the many varied uses for this air. I will suggest only those that I have had experience with during the last two years. There may be many more advantageous uses for it, but I leave them for others to demonstrate. All I wish is to show its value to us whether it be for the laboratory, for the operating-room, or for the extracting-room.

Preparing and filling of sensitive cavities with plastic filling-materials becomes an easy task when the cavity is thoroughly dry; a stream of compressed air will dry the cavity and will enable the operator to do better and quicker work. Let us take a large buccal cavity in a lower molar, involving, say, middle and gingival thirds occluso-gingivally and the middle-third mesio-distally. These, we will all agree, are by no means easy to fill as they should be filled, especially so in cases where one has to do it without the use of rubber dam. Such submarine fillings are never satisfactory. A current of air applied for the period of two minutes will, in most mouths, entirely dry the seat of operation, and after the filling is inserted it will require some effort on the part of the patient to bring about the normal moisture.

In preparing any and all cavities, we are obliged to stop frequently and blow out the chips obscuring the point of operation, and not infrequently do our patients complain of heat generated by a rapidly revolving bur. Let us take a very small rubber tube, the diameter of the opening of which need not be over one-thirty-second of an inch, and wire it around the engine cable down to the point of the hand-piece; now turn on your air-pressure and go ahead with the drilling. The current of the air will not only keep the point of operation in constant sight and free from chips, but will also prevent the bur from heating and thereby will, necessarily, lessen pain and duration of operation. Nowadays we are after modes and means calculated toward lessening degree and amount of pain, and toward doing work with expedition, and for this purpose alone the compressed air is a desirable feature. Should it be desired to heat the air before it reaches the cavity, that is also easily accomplished, for all that is necessary is for us to state our needs and wants and there are many ingenious minds among us to devise such instruments and accessories as may be needed and are not yet in the market.

In taking impressions and bites in wax or gutta-percha, or in various modelling compounds requiring heat for their plasticity, all one has to do after their introduction into the mouth is to turn on the compressed air and cool off the mass in a minute. You will have a sharper impression or bite as the result, and the patient will not get tired out and spoil the impression by involuntarily closing the mouth or changing the relative position of the jaws in case of bite. Again, you are saving time, and the trouble of preparing another "hot cake" is obviated.

In treatment of empyema of the maxillary sinuses, or of a wound after a root incision, or of any other injury about the mouth, where the parts to be treated are remote from sight and easy reach of the operator, and where a thorough medication is required, whether it be of a liquid or of a powder nature, here again the best mode of application is easily solved. If it be a liquid, a pointed atomizer point will, by means of gentle but steady pressure of air, do the work more effectively than when applied by old methods. If it be some powder medicament, I would use a glass pipette into which I gather a few pinches of this powder; the free end of this pipette is attached to the air valve and the opposite end is inserted just at the opening of the wound. Then in the language of kodakers, "you touch the button, and it does the rest." The powder so applied will be distributed all over the wound more uniformly; the deepest portions being reached will quickly stimulate healthy granulations. These deepest portions, as a rule, are slighted, owing to inaccessibility of the parts; but here the force can be exerted as the case may require; there is no danger of the powder so distributed coming back into the operator's face, as in the case of a clown in Barnum's circus, who, upon being instructed to give some pulverized medicament to a sick donkey, made a large cardboard pipe and filled it with the prescribed powder. Then upon inserting one end of the pipe into the donkey's mouth and the other end into his own, he took a long breath preparatory to blowing the powder into the donkey's mouth, but the donkey's olfactory nerve became tickled and he blew first! It is needless to say that the attempted operation was not a success. Had the clown used the compressed-air outfit all would have gone well.

In treating pyorrhœa in all its varieties and stages, and in many diseases of the pericementum, one saves much time and effects a permanent cure considerably quicker by the use of a compressed-air atomizer and treating the diseased parts directly by daily spraying with proper medicaments. Marshall, in his work on "Operative Dentistry," suggests the use of atomizers for this purpose.

Here again the compressed-air apparatus enables one to send uninterrupted streams as strong or as weak as the case in hand may demand into the deep tissues, down into pus-pockets where it is most needed.

To deodorize malodorous mouths before commencing an operation is a desirable feature beyond dispute; here again comes handy the valuable and efficient use of compressed-air.

While touching upon deodorization, I wish to remark that not infrequently our operating-rooms have peculiar "dental odors" about them. Iodoform, carbolic acid, creosote, oils of cassia, cloves, and other essential oils and highly volatile substances, all added to the office, in the mind of many sensitive patients, still more repugnancy than they already have from fear. It is my habit to spray the room with a weak solution of spirits of lavender; fumigate, so to speak, the entire breadth and length of the operating-room. Compressed air does this admirably, and the room smells sweet and fresh and the air is by far more agreeable.

How many bridges and crowns have to be reset in our practice, and all because, in the majority of cases, they were put on while saliva was running freely in and about the abutments? With a steady compressed-air current I can dry up the mouth so that a bridge or a crown can be set under absolutely dry conditions, thereby assuring their stability.

Have you a long soldering job? Here also take your compressed-air hose and attach to it your blow-pipe, and the absolute ease with which the soldering can be done is surprising. Furthermore, no checking of porcelain is apt to occur when the heat, as in this case, is steadily applied.

After giving nitrous oxide or any other general anæsthetic, the patient is brought to quickly by application of a strong force of compressed-air directed squarely toward his face.

Very often patients faint at extraction; compressed air, applied as above, revives them at once.

The use of local anæsthetics by means of injections with the hypodermic needle is objectionable to many patients; still they will not object if you "freeze the gums" by the local abstraction of heat, directing finely divided streams of ether or rhigolene or other such remedy by means of the compressed-air apparatus. Here also it is by far more effective than atomizers operated by hand or foot bellows. The stream is steady, and consequently less of the drug is used and the required numbness is obtained in much less time.

After extracting, I often check the hemorrhage by spraying into the alveoli some suitable styptic; here again I use the compressed-air outfit.

The problem of interesting the children, while doing some painful operation for them, is a hard one; I often let the child help me by holding the compressed-air valve and keeping the cavity blown out. They like the idea of helping you, their mind is away from the seat of trouble, and you accomplish better and quicker work with but an occasional involuntary flinch from them.

No doubt there are many and many more useful ways of utilizing the compressed air, once it be installed in our offices. They become apparent as one uses the apparatus and becomes acquainted with it. When I first commenced to use it, I applied it only where I wanted to prepare a difficult cavity without the use of the rubber dam. Since then, all the other various uses I have mentioned followed naturally, each in its respective turn. I know that but very few have ever used compressed air; many have never thought of it. I wish all would try it and become convinced, and, as I have said before, add more to the already numerous enough list of advantageous uses of compressed air in dentistry to commend its intrinsic value.—*Dental Register.*

